# Executive Functions and Emotion Regulation in Attention-Deficit/Hyperactivity Disorder and Borderline Intellectual Disability

**DOI:** 10.3390/jcm9040986

**Published:** 2020-04-01

**Authors:** Elena Predescu, Roxana Sipos, Cristina A. Costescu, Anamaria Ciocan, Diana I. Rus

**Affiliations:** 1 Department of Neuroscience, Discipline of Psychiatry and Pediatric Psychiatry, Iuliu Hațieganu University of Medicine and Pharmacy Cluj-Napoca, Cluj-Napoca Manastur Street no. 54 C, 400660 Cluj-Napoca, Romania; predescu.elena@umfcluj.ro; 2 Special Education Department, Faculty of Psychology and Educational Sciences, Babes-Bolyai University, Sindicatelor Street no 7, 400029 Cluj-Napoca, Romania; christina.costescu@gmail.com; 3 Department of Clinical Psychology and Psychotherapy, Faculty of Psychology and Educational Sciences, Babes-Bolyai University, Republicii Street no 37, 400015 Cluj-Napoca, Romania; anamaria.ciocan.ac@gmail.com (A.C.); rus.dianaioana@gmail.com (D.I.R.)

**Keywords:** emotion regulation, executive function, behavioral/emotional problems, ADHD, borderline intellectual disability

## Abstract

The main objective of this study is to investigate the multiple relations and to determine the differences between executive functions (EFs), emotion regulation, and behavioral and emotional problems in children with attention-deficit/hyperactivity disorder (ADHD), borderline intellectual disability (ID), and typical development (TD). The sample included 85 children aged 6 to 11 years, 42 with typical development (TD), 27 with ADHD, and 16 with borderline ID. The results emphasized a positive correlation between adaptive emotion regulation strategies and EFs, and no significant relations between the maladaptive emotion regulation strategies and EFs. In addition, the executive function of planning correlated negatively with anxiety, ADHD symptoms, and conduct problems. The performance of both clinical groups regarding EFs was significantly lower than that of the TD group, and they differed significantly from each other only on visual attention. The presence of oppositional-defiant and conduct problems was higher in both clinical groups than in the TD group, and more anxiety symptoms were reported in children with ADHD. This study supports the idea that emotion regulation, Efs, and clinical symptoms are interconnected. It also profiles the deficits in cognitive functioning and emotion regulation in two clinical groups, thus helping future intervention programs.

## 1. Introduction

One of the key concepts in developmental psychopathology is that of executive dysfunction. Several developmental disorders have been associated with deficits in executive functions (EF), including attention deficit and hyperactivity disorder (ADHD) [1] and intellectual disability (ID) [2]. Usually, the EF role is considered separately for each of these disorders, and there have been attempts to describe different types of EF problems. These attempts pointed to the need for a better understanding of the executive functions’ normal development. Cognitive functioning and psychopathology are closely linked to child development. The school years are characterized by cognitive skills and self-regulation strategies refinement while, in some children, clinical symptoms emerge. A disruption in one area of development may be accompanied by impairment in other areas [3].

Emotion dysregulation is also common in individuals with neurodevelopmental disorders, including ADHD and ID. Emotion regulation is a component of the broader concept of self-regulation. Diamond (2013) claims that self-regulation “refers primarily to control and regulation of one’s emotions and overlaps substantially with inhibitory control”, an important component of executive functions (EFs) [4,5]. Some studies highlighted that emotion dysregulation is an important risk factor for aggressive behavior, anxiety symptoms, and eating pathology [6,7]. Also, it appears that children with different neurodevelopmental disorders, like ADHD or ID, have poor emotion regulation skills [8].

Theories on ADHD suggest that deficits in EFs are at the core of the ADHD-syndrome and play a key role in explaining the problems that children with ADHD confront in daily life [9,10]. Evidence suggests that impairments in EF are related to deficits in attention, hyperactivity, impulsivity [11,12], and other associated problems.

When it comes to intellectual disability (ID), emotion dysregulation has received little attention. Individuals with ID may experience deficiencies in the awareness and understanding of their emotional experience, and possibly an increased predisposition to rigidly adhere to a specific self-regulatory response (e.g., aggression or self-harm behaviors) [13]. They tend to utilize a limited range of coping strategies when emotionally aroused [14]. One study revealed that children with ID used more social coping and less goal-directed strategies than typically developed (TD) children during a frustrating task. Therefore, children with ID experienced higher levels of frustration [15]. 

One of the most influential taxonomies of EFs is that proposed by Miyake et al. (2000) [16,17,18]. They support the following three basic EFs: mental set shifting (‘‘Shifting’’), information updating and monitoring (‘‘Updating’’) (operations of working memory), and inhibition of prepotent responses (‘‘Inhibition’’). Diamond (2013) published an EF model in her review on EFs, enforcing the three core EFs proposed by Miyake et al. (2000) and stating that this core EFs contribute to the higher-level EFs: reasoning, problem-solving and planning [4].

In ADHD, clinicians and researchers reported poor inhibition [19,20] and impairment in attention and time reproduction [20] comparative to controls. Findings regarding working memory (WM) deficits in ADHD are inconsistent. A study that investigated which parts of WM may be affected in ADHD found that both visuospatial short-term memory (STM) and central executive were impaired aside from a motivational deficit [21]. On the other hand, results claiming no impairment in WM in ADHD are also present in literature [19,20]. 

Research also documents a clear EFs impairment in ID. Children with ID display deficits in WM, auditory sustained attention, visual selective attention, visual categorization, inhibition, and planning when compared to the performance of a mentally age-matched group [22,23,24]. In addition, WM deficits increased with the degree of ID [22].

The relation between emotion regulation, EFs, and behavioral problems has received little attention. Having deficits in emotion regulation is an important risk factor for conduct problems [6]. Self-regulation and, implicitly, emotion regulation influence planning abilities [4]. Planning has been related to performance in the social domain [25] and aggression [26]. 

The main objective of our study is to investigate the multiple relations and to determine the differences between EFs, emotion regulation, and the behavioral/emotional problems in children with ADHD, borderline ID, and typical development (TD). Within this objective, we aim to test a mediation model, considering that the relationship between emotion regulation and conduct problems is mediated by planning, a high-order EF. Most studies on EFs in neurodevelopmental disorders have focused on the core EFs, but we assessed other EFs, such as visual attention, planning, and STM (as a component of WM). Unlike EFs, emotion regulation in ADHD has been the subject of few studies. The research on EFs and emotion regulation in children with borderline ID is scant. Moreover, we used a multi-method approach for assessing emotion regulation (parent-reports and an observational scale), thus making possible a more comprehensive assessment of the concept. 

## 2. Experimental Section

### 2.1. Participants

We conducted a prospective study on 85 children aged 6 to 11 years (M = 9.12, SD = 1.41), 42 typically developed (TD) children (M = 9.16, SD = 1.01) and 43 diagnosed with attention-deficit/hyperactivity disorder (ADHD) or borderline intellectual disability (ID; IQ ranging from 70 to 85; M = 9.07, SD = 1.72). The TD children sample included 22 girls and 20 boys, recruited from a public school in Gherla. Twenty children were first-graders and 22 were third graders. The clinical groups included 27 children diagnosed with ADHD and 16 diagnosed with borderline ID, without comorbidities, diagnosed via the 10th revision of the International Statistical Classification of Diseases and Related Health Problems (ICD 10)-based clinical interviews. Twenty-four children were recruited from special education schools, and 19 children were recruited from the Clinic of Pediatric Psychiatry from Cluj-Napoca. 

### 2.2. Instruments

Child Behavior Checklist (CBCL) [27]. CBCL is a valid and reliable tool used to assess children’s emotional and behavioral problems and should be filled in by the parent/caretaker who spends the most time with the child. It assesses various problems and child functioning over the past 6 months. CBCL has 113 items reported on a 3-point Likert Scale. The answers are grouped into seven domain-specific scales. This questionnaire showed good validity and reliability coefficients and is adapted for the Romanian population. 

Emotion Regulation Checklist (ERC) [28]. ERC is a 24-item instrument developed to assess the children’s emotional regulation level. It has two scales, Emotion Regulation (ER; 8 items—high scores indicate a good capacity of emotion modulation) and Emotional Lability/Negativity (L/N; 16 items—high scores show an excessive emotional reactivity and a frequent mood change). ER assesses the emotional expression, empathy, and emotional self-awareness, and L/N assesses the lack of flexibility, anger dysregulation, and mood lability. Items are rated on a 4-point Likert scale (1—almost always to 4—never). The questionnaire was translated into Romanian using the study conducted by Molina et al. [29]. 

Child Adjustment Scale [30]. The Child Adjustment Scale is a 33-item measure of the child’s socioemotional adjustment. Each item is rated on a 5-point Likert scale (1—almost never to 5—almost always). It includes four subscales: peer relations, work habits, compliance, and emotional health.

NEPSY: A developmental neuropsychological assessment [31]. NEPSY assesses the neuropsychological development of children aged 3 to 12 years [32]. It has good concurrent and predictive validity test–retest reliability for the subscales. The internal reliability coefficient is r = 0.80. NEPSY differentiates the atypical development profiles from the typical ones and is adapted and validated for the Romanian population. The subscales used in this study were attention and executive functioning (visual attention and tower), visuospatial processing (block construction), and memory and learning (delayed memory for faces). The total administration time for these subscales was approximately 30–40 min for each child.

- Visual attention domain measures visual attention speed and accuracy [16]. The children’s task is to scan a linear array of pictures and to mark the targets as quickly and accurately as possible. For school-aged children, we used the cats and faces targets.
- Tower subtest is a task that assesses a series of executive functions such as planning, monitoring, problem-solving, and self-regulation [16]. The children must move the three colored balls to target positions, on three pegs, following some rules. The task is time-limited (30–60 s), and its difficulty varies according to the number of moves and complexity.
- Block construction is a visuospatial processing task that requires children to build constructions using a different number of blocks. This task has a medium level of difficulty, and every subset has a time limit of 30 to 60 s.
- Delayed faces memory assesses children’s memory capacity. Children are shown 16 pictures of boys and girls, and they must memorize them. After 30 minutes, the experimenter shows the children 16 sets of pictures, each containing three pictures, and the children’s task is to recognize the person that they have seen before from every set of pictures. During the delayed memory task, children must hold in mind for several minutes some visual information. We considered the delayed memory task a measure of short-term memory (STM).

Tower construction task (Anger-eliciting task—observational scale) [33]. The children’s task is to build a wooden 10-block tower. A picture of a tower is first presented, then the children are instructed to build a tower exactly like the one in the picture within 2 min and 40 s in order to receive a reward (a candy in our study). The task is impossible to solve because two blocks are slightly rounded on one side. At the end of the assessment, the children are explained the impossibility of the task. It is an anger-eliciting task developed to assess the anger regulation strategies using a behavioral observation scale. Both the anger-eliciting task and the behavioral observation scale were developed and validated by Rohlf and Krahe [33]. The observational scale includes three categories of emotion regulation strategies: adaptive (solution orientation, substituting the anger expression, and verbalizing the cognitive strategies), maladaptive (visual focus, verbal focus, anger expression, resignation), and neutral strategies (ineffective help-seeking). 

Raven’s progressive matrices were used to assessed children’s intellectual abilities. 

A questionnaire was used to collect general information regarding children’s age, educational level, clinical diagnosis, and other demographic data. 

### 2.3. Procedure

We used a quasi-experimental design to analyze the associations between children’s characteristics (TD children and children diagnosed with ADHD or borderline ID), adaptive or maladaptive emotional regulation strategies, and executive functions. The study was carried according to the law concerning the conduct of clinical trials, including abidance by international ethical standards foreseen in the updated Helsinki Declaration of Human Rights. We obtained the approval of the local ethics committee to conduct the study. Data were used respecting the regulation regarding the privacy and subject’s identity protection, and informed consent was obtained for each participant. All questionnaires were filled in by the parents. Children that agreed to participate in the study were assessed in a 30–40-minute individual session. The TD children were assessed in a school laboratory and the children diagnosed with ADHD or ID, in a playroom used for psychological assessments. The NEPSY battery tasks were completed first (visual attention task, the learning task from the delayed memory subtest, the tower and block construction). The anger-eliciting task was applied next. After receiving the instructions, behaviors and the involvement level were coded using the observational scale [33]. Every child was rewarded after completing the task. 

Data were analyzed using SPSS 20. Pearson correlation was used to test the associations between the studied variables (emotional regulation, cognitive functioning, emotional and behavioral problems) for the entire sample, and separately for two age groups (7 to 9 and 9 to 11 years). Independent *t*-test was used to compare the studied variables between the clinical groups (ADHD and borderline ID) and the non-clinical group (TD). 

## 3. Results

### 3.1. Differences in Cognitive Functioning, Emotion Regulation, and Behavioral and Emotional Problems Across Diagnoses

Means (M) and standard deviations (SD) were computed for the cognitive functions, emotion regulation, and behavioral and emotional problems in the typical development (TD) and clinical groups (see Table 1). A *t*-test for independent samples was used to investigate the differences between the TD children and those diagnosed with ADHD or borderline ID.

As seen in Table 1, the TD group scored better than the clinical groups on every assessed cognitive function. Generally, the means differences were important and similar between the TD group and the clinical groups, but for visual attention, they followed a different pattern. The mean score was higher in the TD group (M = 8.95, SD = 1.03) than in both ADHD (M = 7.45, SD = 5.23) and borderline ID groups (M = 3.81, SD = 2.00), but the difference was significant also between the clinical groups. The visuospatial processing mean in TD children (M = 14.04, SD = 2.54) was higher than the means for ADHD group (M = 9.17, SD = 3.82) and borderline ID group (M = 7.06, SD = 2.56), but the differences did not reach significance. Regarding the emotion regulation strategies, the ADHD group reported a higher use of maladaptive strategies (M = 3.46, SD = 2.06) than the TD group (M = 2.83, SD = 2.25) and borderline ID group (M = 2.00, SD = 1.47). Adaptive strategies were more frequently used by the TD group (M = 5.07, SD = 2.64) than the clinical groups which reported similar frequencies. When analyzing the behavioral/emotional problems reported on CBCL, we noticed a higher level of affective problems, anxiety, oppositional-defiant, and conduct problems reported for the children in the ADHD group. The borderline ID group scored higher on ADHD and conduct problems than the children from the TD group, but lower than the ADHD group. 

TD group scored higher on cognitive functions than both ADHD and borderline ID groups, the difference being significant for visual attention, memory, and planning. The difference was not significant for visuospatial processing (see Table 2). When comparing the TD group with the ADHD group, significant differences were registered for visual attention (t(64) = 1.79, p = 0.00), memory (t(64) = 5.73, p = 0.006), and planning (t(66) = 5.11, p = 0.00). Similar results were registered for the comparison between TD group and borderline ID group for visual attention (t(56) = 12.81, p = 0.00), memory (t(56) = 4.73, p = 0.047), and planning (t(56) = 4.92, p = 0.001).

A *t*-test was used to assess the differences in emotion regulation strategies across groups. No significant differences were registered (see Table 2). The adaptive emotional regulation strategies mean was higher in the TD group (M = 5.07, SD = 2.64) than in both ADHD (M = 4.53, SD = 3.09) and borderline ID groups (M = 4.50, SD = 2.71), but the differences did not reach significance. The maladaptive emotional regulation strategies mean in TD children (M = 2.83, SD = 2.25) was smaller than the mean for ADHD group (M = 3.46, SD = 2.06) and higher than the mean for the borderline ID group (M = 2.00, SD = 1.47), but the differences did not reach significance. Children included in the clinical groups (ADHD and borderline ID) reported higher levels of emotional and behavioral problems on CBCL, than the TD children (see Table 1). Significant differences were registered for oppositional-defiant problems between TD children (M = 2.33, SD = 2.19) and the children from the ADHD group (M = 4.29, SD = 3.67), t(67) = −2.78, *p* = 0.00. Similar results were obtain for the difference between TD group and borderline ID group (M = 2.62, SD = 2.96), t(56) = −4.10, *p* = 0.036. Significant differences were registered also for conduct problems between TD children (M = 1.78, SD = 2.64) and children with ADHD (M = 5.66, SD = 6.33), t(67) = −3.52, *p* = 0.00, and between TD children and those with borderline ID (M = 3.50, SD = 4.84), t(56) = −1.72, *p* = 0.00. For the ADHD problems reported by the parents on CBCL, a significant difference t(67) = −2.89, *p* = 0.00 was registered between TD children (M = 4.14, SD = 3.15) and those with ADHD (M = 6.96, SD = 4.94). The ADHD group reported more anxiety problems (M = 3.11, SD = 2.81) than the TD group (M = 2.28, SD = 2.07), and the difference was significant, t(67) = −1.39, *p* = 0.02.

No significant differences were registered between the ADHD group and borderline ID group for cognitive function visuospatial processing, memory and planning, the adaptative and maladaptive emotion regulation strategies, and emotional and behavioral problems measured by CBCL (see Table 3). The only significant difference between the clinical groups was registered for visual attention. The ADHD group mean (M = 7.45, SD = 5.23) was higher than that of borderline ID group (M = 3.81, SD = 2.00), the difference being significant t(38) = 2.65, *p* = 0.002.

### 3.2. The Relation between Emotion Regulation, Cognitive Functioning, Emotional and Behavioral Problems 

When considering the whole sample, we found significant correlations between the cognitive functions assessed and the emotion regulation strategies measured by the observational scale. The executive functions correlated positively with the adaptive emotion regulation strategies as a unitary concept. The correlation between the adaptive emotion regulation strategies and visual attention was r(69) = 0.256, *p* = 0.034. The correlations were also significant with delayed memory r(69) = 0.298, *p* = 0.013, planning r(69) = 0.364, *p* = 0.002 and visuospatial processing r(69) = 0.246, *p* = 0.042. The correlations were positive, meaning that a frequent use of adaptive emotion regulation strategies is associated with better cognitive function performances. 

When analyzing the specific adaptive emotion regulation strategies, verbalizing the strategies correlated positively with visual attention r(69) = 0.383, *p* = 0.001, planning r(69) = 0.322, *p* = 0.007, delayed memory r(69) = 0.267, *p* = 0.026, and visuospatial processing r(69) = 0.306, *p* = 0.011. Substituting the anger, another adaptive emotion regulation strategy, correlated positively with both visual attention r(69) = 0.279, *p* = 0.020 and visuospatial processing r(69) = 0.352, *p* = 0.003. 

No significant correlations were found between the maladaptive strategies as a unitary concept and the cognitive functions assessed. This may be explained partially by the fact that verbal focusing correlated positively with visual attention r(69) = 0.239, p = 0.047, whereas resignation correlated negatively with all cognitive functions assessed, ranging from −0.359 with delayed memory to −0.238 with planning, meaning that a lesser use of resignation is associated with better performances on visual attention, delayed memory, planning, and visuospatial processing. (The correlations are presented in Table A1).

When analyzing the relation between emotion regulation reported by the parents [30] and the cognitive functions assessed, we found significant correlations between emotion regulation and visual attention r(69) = 0.528, *p* = 0.000, delayed memory r(69) = 0.270, *p* = 0.025, and visuospatial processing r(69) = 0.513, *p* = 0.000, but no significant correlation between emotion regulation reported by parents and planning r(69) = 0.181, *p* > 0.05. 

Analyzing the possible relationship between the cognitive functions assessed and emotional/behavioral problems as measured by CBCL, planning correlated negatively with anxiety problems r(84) = −0.235, *p* = 0.031, ADHD problems r(84) = −0.221, *p* = 0.043 and conduct problems r(84) = −0.276, *p* = 0.011. Delayed memory also correlated negatively with conduct problems r(84) = −0.237, *p* = 0.032. 

We analyzed the associations between cognitive functions, emotion regulation strategies, and emotional/behavioral problems for the age-based groups (7 to 9 and 9 to 11 years). For the 7 to 9 years group, a positive correlation was found between adaptive emotional regulation strategies (as a unitary concept) and planning r(20) = 0.509, *p* = 0.022. Negative correlations were noted between substituting the anger (an adaptive emotion regulation strategy) and visual attention r(20)= −0.462, *p* = 0.040, and between visual attention and affective problems measured by CBCL r(20) = −0.581, *p* = 0.007, meaning that, in this age-group, having poor executive function is associated with a higher level of affective problems. For the 9 to 11 years group, delayed memory correlated with the adaptive emotion regulation strategies r(22) = 0.595, *p* = 0.003. Maladaptive emotion regulation strategies (as a unitary concept) correlated negatively with planning r(22) = −0.473, *p* = 0.026, meaning that, in this age-group, having poor executive function is associated with the use of maladaptive emotion regulation strategies. Moreover, maladaptive emotion regulation strategies correlated positively with conduct problems measured by CBCL r(22) = 0.482, *p* = 0.023. We did not find any significant correlation between the reported emotion regulation and other variables. (The correlations are presented in Table A2).

### 3.3. Testing the Relationship between Maladaptive Strategies, Planning, and Conduct Problems 

The model tested had the executive function of planning as a mediator in the relation between maladaptive emotion regulation strategies and conduct problems measured by CBCL. Statistical analysis showed a positive correlation between maladaptive emotion regulation strategies and conduct problems r(69) = 0.245, *p* = 0.04. The executive function of planning, as a mediator, showed a correlation with maladaptive emotion regulation strategies r(69) = 0.099, *p* = 0.41 that did not reach significance and a significant negative correlation with conduct problems measured by CBCL r(84) = −0.276, *p* = 0.01. Thus, the preliminary conditions for demonstrating mediation were not met, and the mediation analysis could not be performed.

## 4. Discussion and Conclusions

The main objective of our study is to profile the deficits in cognitive functioning (executive functions, EFs) and emotion regulation for two neurodevelopmental disorders: ADHD and borderline ID. Both clinical groups scored significantly lower on EFs than the TD group. The attention impairment reported for the ADHD group is consistent with other study results [20]. For the impairment in planning, the findings are conflicting [34,35], but our results support the hypothesis that planning is impaired in ADHD. The ADHD group deficit in short term memory (STM) has also been identified in other studies [21]. For the children with borderline ID, the identified impairments in attention and planning are consistent with the findings from earlier studies. [22,23,24]. A poor working memory (WM) performance in children with borderline ID has been previously described [22], but our results suggest that a WM component is affected. Previous studies have also found specific subgroups of children with ID to have broad deficits in working memory functioning [36,37]. Henry et al. (2001) found the subgroup of respondents with borderline ID to have a specific impairment in the phonological subsystem [38]. Schuchardt et al. (2010) concluded in their study that 15-year-old children with ID registered deficits on all measures of the central executive, the visual–spatial sketchpad, and the phonological loop administered. The deficits increased with the degree of ID, the average-ability students having better WM performance than their chronological peers with borderline ID. These results suggest a general dysfunction in WM depending on the intelligence level [22]. In our study, no significant differences were found between the three groups in visuospatial processing performance.

In terms of emotion regulation, the comparisons between the three groups did not reveal any significant difference, but the children with ADHD reported higher use of maladaptive strategies than the TD and borderline ID children. Adaptive strategies were more frequently used by TD children, the clinical groups reporting lower, but similar frequencies. 

The presence of oppositional-defiant and conduct problems was higher in the ADHD and borderline ID groups than in the TD group. Planning correlated negatively with ADHD and conduct problems. These findings suggest that clinical group behavioral problems may be a result of poor executive functioning and not of poor emotion regulation.

As expected, the ADHD symptoms were more prevalent in the ADHD group than in the TD group. In addition, children diagnosed with ADHD reported more anxiety and affective symptoms than the TD children. There are studies on ADHD comorbidities suggesting that in cases with comorbidities, there is also a greater involvement of large neural networks. In the Sonuga–Barke dual-pathway model, the individual’s executive functions are divided into the traditional executive functions or cold executive functions, such as attention, working memory, planning, and inhibition, and hot executive functions which are related to neuropsychological processes such as emotion and motivation [39]. The distinction between cool (cognitive modulation of information) and hot EFs linked to emotional self-regulation and the reward system might underlying such different traits. It can be essential for effective interventions to identify behavior/emotional risk factors in these children [40,41,42]. 

The two clinical groups differed from each other only in regard to visual attention; the ADHD group performing better than the borderline ID group. Visual attention is defined as the mechanism by which we select visual information relevant to everyday behavior [43]. Evidence suggests that attention allocation may support working memory [44,45]. Also, some studies have shown a strong relationship between attention, eye movements, and working memory, implying that attention influence not only what people experience and remember but also what people attend to [46]. According to these findings, there is a strong correlation between seeing and remembering. In a study on children with ADHD and TD children, a qualitative analysis of the protocol of eye-tracking indicated that 80% of the children with ADHD showed a typical fuzzy eye-tracking behavior, while 80% of the TD children showed a typical serial eye-tracking behavior. The quantity of the fixation length was the same in ADHD and TD children, but the quality of the fixation had been very different; children with ADHD showing a discontinuous and uncoordinated attention system [47]. In our study, the two clinical groups differed significantly from each other only on visual attention; for memory and planning, the results were almost similar, and for the visuospatial processing, the ADHD group performed better, but not significantly better. A possible explanation may reside in the discontinuous and uncoordinated attention system of the children with ADHD, making them less performant in memory and planning even if they had superior visual attention and visuospatial processing when compared with the children with borderline ID. Another explanation may reside in the lower motivation and higher oppositional-defiant problems or impulsivity, which may also alter the performance. Both clinical groups performed significantly poorer than the TD group, emphasizing that impairment in attention is not specific only for ADHD and also indicating that this executive function deficit can be even more pronounced in other neurodevelopmental disorders such as borderline ID. Attention deficit, which is often seen in children with ID, significantly affect their learning and behavior [48,49]. A study on visual attention of children with ADHD reported significantly impaired sustained attention and visual processing speed but intact attentional selectivity, perceptual threshold, and visual short-term memory capacity, supporting the notion of different impairment of attentional functions in children with ADHD [50].

Another goal of this study is to investigate the relations between EFs, emotion regulation, and emotional and behavioral problems as measured by CBCL. The results on the observational scale emphasized a positive correlation between adaptive emotion regulation strategies (as a unitary concept) and each measured EFs: visual attention, planning, and delayed memory or STM (as a component of WM). Further, we analyzed each adaptive emotion regulation strategy in relation to EFs. Verbalizing the strategies positively correlated with all EFs and substituting the anger positively correlated only with visual attention, reinforcing the common belief that adaptive emotion regulation strategies associate with better EF performances. 

The unitary concept of maladaptive emotion regulation strategies, measured by the observational scale, did not relate to EFs. This situation is partially explained by the fact that one maladaptive strategy correlated negatively with all EFs, whereas another one correlated positively with visual attention. For the emotional adjustment reported by parents, we found a positive relation between emotion regulation and both visual attention and delayed memory (STM). These findings suggest that children with good executive functions may use both adaptive and maladaptive emotion regulation strategies, but children with poor executive functions are more prone to use maladaptive emotion regulation strategies, such as resignation. 

The examination of the relation between EFs and emotional/behavioral problems revealed that planning correlated negatively with anxiety, ADHD symptoms, and conduct problems. These findings are consistent with previous studies showing that a deficit in planning can be related to behavioral problems [26]. In addition, our results indicated that planning can also be related to emotional problems. Recent studies indicate that hyperactive children can find it more difficult to positively regulate their emotions [51], but our results indicate more possible explanations for that, the children with ADHD reporting higher use of maladaptive emotion regulation strategies and also a higher level of anxiety and affective problems. 

Differences regarding the relations between EFs, emotion regulation, and emotional/behavioral problems were observed between the two age groups. The younger group (aged 7 to 9 years) showed a positive correlation between adaptive emotion regulation strategies (as a unitary concept) and planning. The adaptive emotion regulation strategy of substituting the anger correlated negatively with visual attention. In the older group (aged 9 to 11 years), maladaptive emotion regulation strategies (as a unitary concept) correlated negatively with planning and positively with conduct problems. 

Moreover, we did not find support for our mediation model. The executive function of planning did not mediate the relation between maladaptive emotion regulation strategies and conduct problems. Even though the Diamond’s (2013) model of EFs suggests that emotion regulation influences the higher executive functions, such as planning [4], we did not find any significant relation between maladaptive emotion regulation strategies and planning. Interestingly, this relation reached significance for the 9 to 11 years age group, where maladaptive emotion regulation strategies (as a unitary concept) correlated negatively with planning, but not in the entire sample. Our sample included children with ages between 6 and 11 years, and this may explain the result. 

This study has several limitations. The clinical sample size was relatively small, primarily because we included only patients diagnosed with ADHD or borderline ID without other diagnosed comorbidities. Another important limitation is that our sample had a wide range of ages. As we noticed, there were differences between the two age groups regarding the relationship between emotion regulation and EFs, and this fact might have influenced the findings for the entire sample. Even though our study is a complex one and tries to cover almost all the executive functions mentioned by Diamond (2013) [4] in her model and tries to investigate the possible connections with emotion regulation skills and emotional and behavioral problems, for a scientific validation of our theoretical model, a bigger sample size is needed. Therefore, future studies should consider more participants for a better generalization of the results. Also, the investigated outcomes from our research could be measured in various ways, which may better explain the interconnection between several factors. For example, future studies should also take into consideration tasks for children measuring emotion regulation skills and emotional problems. In this way, the confound variables, such as the parents’ perception, could be better controlled. Considering all the above-mentioned limitations, we recommend interpreting the results of this work with caution.

The study supports the idea that emotion regulation, EFs, and clinical symptoms are interconnected. Moreover, it suggests that behavioral problems in the clinical groups may be due to poor executive functioning and not a result of poor emotion regulation. Finally, it brings to light new aspects regarding cognitive functioning, emotion regulation, and behavioral and emotional problems in ADHD and borderline ID. A better understanding of each neurodevelopmental disorder can lead to the advancement of more targeted intervention programs. 

## Figures and Tables

**Table 1:** Means (M) and standard deviations (SD) for cognitive functions, emotion regulation strategies, and behavioral/emotional problems measures.

		M (SD) TD (*n* = 42)	M (SD) ADHD (*n* = 27)	M (SD) Borderline ID (*n* = 16)
Cognitive functions	Visual attention	8.95 (1.03)	7.45 (5.23)	3.81 (2.00)
	Memory	12.16 (2.15)	8.33 (3.27)	8.81 (3.01)
	Planning	12.92 (2.78)	7.46 (5.98)	7.68 (5.27)
	Visuospatial processing	14.04 (2.54)	9.17 (3.82)	7.06 (2.56)
Emotion regulation	Maladaptive strategies	2.83 (2.25)	3.46 (2.06)	2.00 (1.47)
	Adaptive strategies	5.07 (2.64)	4.53 (3.09)	4.50 (2.71)
Behavioral/emotional problems	Affective problems	3.26 (3.54)	4.59 (3.93)	3.68 (4.20)
	Anxiety	2.28 (2.07)	3.11 (2.81)	2.81 (2.80)
	Somatic problems	0.71 (1.33)	0.51 (1.01)	0.75 (1.61)
	ADHD	4.14 (3.15)	6.96 (4.94)	5.68 (3.94)
	Oppositional-defiant	2.33 (2.19)	4.29 (3.67)	2.62 (2.96)
	Conduct problems	1.78 (2.64)	5.66 (6.33)	3.50 (4.84)

TD, typical development; ADHD, attention-deficit/hyperactivity disorder; ID, intellectual disability.

**Table 2:** Differences between TD, ADHD, and borderline ID children.

			*t*-test
		TD versus ADHD	TD versus Borderline ID
Cognitive functions	Visual Attention	t(64) = 1.79, *p* = 0.00	t(56) = 12.81, *p* = 0.00
	Memory	t(64) = 5.73, *p* = 0.006	t(56) = 4.73, *p* = 0.047
	Planning	t(66) = 5.11, *p* = 0.00	t(56) = 4.92, *p* = 0.001
	Visuospatial processing	t(57) = 5.71. *p* = 0.09	t(56) = 9.31, *p* = 0.572
Emotion regulation	Maladaptive strategies	t(55) = −0.95, *p* = 0.907	t(52) = 1.20, *p* = 0.205
	Adaptive strategies	t(55) = 0.695, *p* = 0.06	t(52) = 0.713, *p* = 0.328
Behavioral/ Emotional problems	Affective problems	t(67) = −1.45, *p* = 0.648	t(56) = −0.388, *p* = 0.363
	Anxiety	t(67) = −1.39, *p* = 0.02	t(56) = −0.781, *p* = 0.732
	Somatic problems	t(67) = 0.65, *p* = 0.188	t(56) = −.086, *p* = 0.450
	ADHD	t(67) = −2.89, *p* = 0.002	t(56) = −1.55, *p* = 0.097
	Oppositional-defiant	t(67) = −2.78, *p* = 0.00	t(56) = −0.410. *p* = 0.036
	Conduct problems	t(67) = −3.52, *p* = 0.00	t(56) = −1.727, *p* = 0.002

**Table 3:** Differences between children with ADHD and children with borderline ID.

		*t*-test
		ADHD versus Borderline ID
Cognitive functions	Visual Attention	t(38) = 2.65, *p* = 0.002
	Memory	t(38) = −0.46, *p* = 0.65
	Planning	t(40) = −0.12, *p* = 0.32
	Visuospatial processing	t(31) = 1.85. *p* = 0.13
Emotion regulation	Maladaptive strategies	t(25) = 2.06, *p* = 0.09
	Adaptive strategies	t(25) = 0.029, *p* = 0.47
Behavioral/ Emotional problems	Affective problems	t(41) = 0.71, *p* = 0.66
	Anxiety	t(41) = 0.33, *p* = 0.27
	Somatic problems	t(41) = −0.57, *p* = 0.08
	ADHD	t(41) = 0.87, *p* = 0.29
	Oppositional-defiant	t(41) = 1.54, *p* = 0.25
	Conduct problems	t(41) = 1.17, *p* = 0.28

## Appendix A

**Table A1:** Correlation coefficients for the entire sample.

Measurements	1	2	3	4	5	6	7	8	9	10	11	12	13	14	15	16	17	18	19	20
1. Visual attention (NEPSY)	1																			
2. Delayed memory (NEPSY)	0.394 **	1																		
3. Planning (NEPSY)	0.374 **	0.569 **	1																	
4. Visuospatial processing (NEPSY)	0.756 **	0.513 **	0.546 **	1																
5. Visual focusing	−0.051	0.206	0.221	0.037	1															
6. Verbal focusing	0.239 *	0.110	0.080	0.232	0.098	1														
7. Anger expression	0.217	0.058	0.040	0.052	−0.028	0.084	1													
8. Resignation	−0.276 *	−0.359 **	−0.238 *	−0.265 *	−0.076	0.125	0.082	1												
9. Solution orientation	−0.115	0.087	−0.104	−0.111	0.139	−0.064	−0.255 *	−0.431 **	1											
10. Substituting the anger	0.279 *	0.233	0.194	0.352 **	−0.082	0.129	−0.201	−0.141	−0.049	1										
11. Verbalizing the strategies	0.383 **	0.267 *	0.322 **	0.306 **	0.315 **	0.130	0.024	−0.203	0.060	−0.019	1									
12. Maladaptive strategies	0.218	0.104	0.099	0.148	0.329 **	0.762 **	0.622 **	0.280 *	−0.220	−0.075	0.157	1								
13. Adaptive strategies	0.256	0.298 *	0.364 **	0.246 *	0.202	0.053	−0.208	−0.533 **	0.785 **	0.313 **	0.489 **	−0.115	1							
14. Emotion regulation	0.528 *	0.270 *	0.181	0.513 **	0.008	0.143	0.242 *	−0.326 **	−0.106	0.282 *	0.262 *	0.183	0.175	1						
15. Affective problems (CBCL)	−0.030	−0.124	−0.134	−0.134	−0.016	0.155	0.046	−0.056	−0.026	−0.031	−0.030	0.114	−0.035	0.145	1					
16. Anxiety (CBCL)	0.061	−0.169	−0.235 *	−0.052	−0.125	0.156	−0.020	−0.070	−0.029	−0.010	−.053	0.043	−0.030	0.119	0.552 **	1				
17. Somatic problems (CBCL)	−0.084	0.082	−0.047	−0.009	−0.017	−0.231	−0.076	−0.176	0.177	0.060	−0.100	−0.232	0.123	0.136	0.424 **	0.235 *	1			
18. ADHD (CBCL)	−0.096	−0.207	−0.221 *	−0.190	0.001	0.093	0.134	0.103	−0.016	−0.002	−0.071	0.155	−0.038	0.070	0.616 **	0.527 **	0.286 **	1		
19. Oppositional-defiant (CBCL_	0.031	−0.198	−0.198	−0.081	−0.031	0.121	0.238 *	−0.050	−0.025	0.050	−0.091	0.196	−0.023	0.293 *	0.617 **	0.600 **	0.389 **	0.777 **	1	
20. Conduct problems (CBCL)	−0.112	−0.237 *	−0.276 *	−0.156	−0.007	0.206	0.204	−0.023	−0.017	0.085	−0.075	0.245 *	−0.007	0.299 *	0.553 **	0.398 **	0.303 **	0.668 **	0.790 **	1

**Table A2:** Correlation coefficients for the 7–9 and 9–11-years group.

**Measurements 7–9 years**	**1**	**2**	**3**	**4**	**5**	**6**	**7**	**8**	**9**	**10**	**11**	**12**	**13**	**14**	**15**	**16**	**17**	**18**	**19**	**20**
1. Visual attentions (NEPSY)	1																			
2. Delayed memory (NEPSY)	−0.189	1																		
3. Planning (NEPSY)	0.007	−0.215	1																	
4.Visuospatial processing (NEPSY)	0.120	0.094	−0.084	1																
5. Visual focusing	−0.019	0.223	0.186	0.242	1															
6. Verbal focusing	0.000	0.217	−0.384	−0.016	−0.065	1														
7. Anger expression	0.170	−0.011	−0.266	−0.076	0.162	0.034	1													
8. Resignation	0.267	0.162	−0.008	−0.227	−0.090	0.382	0.331	1												
9. Solution orientation	0.029	−0.243	0.397	−0.073	−0.252	−0.411	−0.346	−0.373	1											
10. Substituting the anger	−0.462 *	0.047	−0.175	0.216	−0.305	−0.162	−0.381	−0.178	0.077	1										
11. Verbalizing the strategies	0.137	0.008	0.413	0.135	0.567 **	0.234	−0.032	0.287	−0.008	−0.356	1									
12. Maladaptive strategies	0.106	0.231	−0.369	−0.012	0.274	0.789 **	0.586 **	0.532 *	−0.579 **	−0.402	0.335	1								
13. Adaptive strategies	−0.090	−0.222	0.509*	0.105	−0.046	−0.310	−0.472 *	−0.249	0.814 **	0.286	0.387	−0.498 *	1							
14. Emotion regulation	−0.019	0.021	−0.360	−0.423	−0.065	0.053	0.225	0.092	0.011	−0.196	−0.212	0.142	−0.237	1						
15. Affective problems (CBCL)	−0.581 **	0.167	−0.134	−0.278	−0.138	0.205	−0.036	−0.222	0.195	−0.137	−0.096	0.077	0.048	0.165	1					
16. Anxiety (CBCL)	−0.368	0.305	−0.271	−0.084	−0.144	0.066	−0.169	−0.279	−0.138	−0.126	−0.424	−0.099	−0.245	0.077	0.524 *	1				
17. Somatic problems (CBCL)	−0.097	0.202	−0.037	−0.224	−0.250	−0.364	0.169	−0.146	0.354	−0.009	−0.483 *	−0.270	0.014	0.359	0.487 *	0.409	1			
18. ADHD (CBCL)	−0.123	0.245	−0.065	−0.104	−0.097	−0.311	0.265	−0.117	0.229	−0.003	−0.421	−0.142	−0.015	0.005	0.386	0.465 *	0.732 **	1		
19. Oppositional-defiant (CBCL)	0.062	0.215	−0.074	−0.128	−0.068	−0.173	0.042	−0.304	0.316	0.050	−0.527 *	−0.159	−0.042	0.244	0.299	0.442	0.650 **	0.745 **	1	
20. Conduct problems (CBCL)	0.145	0.152	−0.273	−0.185	−0.220	0.012	−0.039	−0.214	−0.249	0.073	−0.467 *	−0.089	−0.029	0.438	0.016	0.048	0.425	0.208	0.636 **	1
**Measurements 9–11 years**	**1**	**2**	**3**	**4**	**5**	**6**	**7**	**8**	**9**	**10**	**11**	**12**	**13**	**14**	**15**	**16**	**17**	**18**	**19**	**20**
1. Visual attentions (NEPSY)	1																			
2. Delayed memory (NEPSY)	0.318	1																		
3. Planning (NEPSY)	0.091	0.398	1																	
4. Visuospatial processing (NEPSY)	0.313	0.227	0.160	1																
5. Visual focusing	0.042	0.169	0.216	0.251	1															
6. Verbal focusing	0.356	−0.191	0.272	0.161	0.095	1														
7. Anger expression	0.046	−0.090	−0.528 *	−0.458 *	−0.033	−0.084	1													
8. Resignation	0.230	−0.395	−0.174	−0.058	0.292	−0.341	0.102	1												
9. Solution orientation	−0.029	0.475 *	0.213	−0.065	−0.120	−0.114	−0.248	−0.586 **	1											
10. Substituting the anger	0.138	0.118	0.219	−0.007	0.051	0.258	−0.221	0.145	−0.066	1										
11. Verbalizing the strategies	0.245	0.244	0.010	−0.058	−0.102	−0.103	0.172	−0.287	−0.400	−0.328	1									
12. Maladaptive strategies	0.324	−0.203	−0.473 *	−0.099	0.349	0.733 **	0.534 *	0.524 *	−0.344	0.088	−0.040	1								
13. Adaptive strategies	0.126	0.595 **	0.312	0.024	−0.083	−0.093	−0.247	−0.581 **	0.859 **	0.246	0.503 **	−0.318	1							
14. Emotion regulation	0.176	0.351	−0.047	0.308	0.070	−0.099	0.077	0.117	−0.043	0.111	0.247	0.010	0.017	1						
15. Affective problems (CBCL)	−0.051	0.008	0.049	−0.058	0.257	0.179	0.109	0.094	0.002	−0.183	0.310	0.271	−0.077	0.330	1					
16. Anxiety (CBCL)	0.165	−0.020	−0.208	0.285	0.099	0.289	−0.013	0.162	−0.092	−0.255	0.324	0.246	−0.200	0.439 *	0.665 **	1				
17. Somatic problems (CBCL)	0.017	−0.006	−0.131	0.271	0.172	−0.270	−0.115	−0.106	0.058	−0.038	0.237	−0.229	−0.204	0.173	0.212	−0.021	1			
18. ADHD (CBCL)	0.106	−0.087	−0.198	0.178	0.239	0.344	0.048	0.316	−0.051	−0.187	0.355	0.380	−0.158	0.470 *	0.798 **	0.809 **	0.197	1		
19. Oppositional-defiant (CBCL)	−0.032	−0.094	−0.270	0.103	0.341	0.158	0.314	0.154	−0.046	−0.135	0.246	0.407	−0.148	0.481 *	0.811 **	0.624 **	0.334	0.819 **	1	
20. Conduct problems (CBCL)	0.075	−0.106	−0.267	−0.012	0.199	0.247	0.395	0.168	−0.146	−0.064	0.159	0.482*	−0.245	0.507*	0.732 **	0.452*	0.173	0.634 **	0.836 **	1
